# Comparison of Attenuation of Striated Muscle between Postmortem and Antemortem Computed Tomography: Results of a Longitudinal Study

**DOI:** 10.1371/journal.pone.0111457

**Published:** 2014-11-03

**Authors:** Hidemi Okuma, Wataru Gonoi, Masanori Ishida, Go Shirota, Yukako Shintani, Hiroyuki Abe, Masashi Fukayama, Kuni Ohtomo

**Affiliations:** 1 Department of Radiology, Graduate School of Medicine, The University of Tokyo, Bunkyo-ku, Tokyo, Japan; 2 Department of Radiology, Mutual Aid Association for Tokyo Metropolitan Teachers and Officials, Sanraku Hospital, Chiyoda-ku, Tokyo, Japan; 3 Department of Pathology, Graduate School of Medicine, The University of Tokyo, Bunkyo-ku, Tokyo, Japan; Tokyo Medical and Dental University, Japan

## Abstract

**Objective:**

We evaluated the postmortem changes of striated muscle by comparing computed tomography (CT) images obtained postmortem and antemortem in the same patients.

**Materials and Methods:**

We studied 33 consecutive patients who underwent antemortem CT, postmortem CT, and pathological autopsy in our tertiary care hospital between April 2009 and December 2010. Postmortem CT was performed within 20 h after death and was followed by pathological autopsy. Pathological autopsy confirmed the absence of muscular diseases such as amyotrophic lateral sclerosis, muscular dystrophy, myositis, and myasthenia, in all of the patients. The CT attenuation values of four cardiac muscle sites (anterior wall of the left ventricle, left ventricular free wall, posterior wall of the left ventricle, and the ventricular septum) and two skeletal muscle sites (the pectoralis major muscle and the erector spinae muscle) were compared between antemortem and postmortem CT using paired *t* test.

**Results:**

Striated muscle had significantly greater attenuation on postmortem CT than on antemortem CT (*P*<0.001) in all six tissue sites. No significant association was found between postmortem change in the CT attenuation of striated muscle and gender, age, or elapsed time since death.

**Conclusion:**

This is the first longitudinal study to show hyperattenuation of striated muscle on postmortem CT images compared with antemortem CT images in the same patients.

## Introduction

High-resolution imaging modalities such as computed tomography (CT) and magnetic resonance imaging are increasingly being used as adjuncts to traditional forensic methods in postmortem studies [1]–[5]. However, clinical radiologists may experience difficulty interpreting postmortem images because of specific and nonspecific postmortem signs [6]. As guidelines for the diagnosis of postmortem images are now being established worldwide, it is important to understand the normal changes on postmortem CT. The postmortem CT features of several organs have already been described [7]–[11]. To our knowledge, however, there are no reports describing the postmortem changes in CT attenuation of striated muscle. Therefore, we conducted a quantitative study in which we compared the attenuation of striated muscle between postmortem and antemortem CT in patients who died in hospital from nontraumatic causes. The results revealed significant difference in the attenuation of striated muscle between postmortem and antemortem CT.

## Materials and Methods

### Study design and donors

The Research Ethics Committee of the University of Tokyo Hospital approved this study, which was conducted in accordance with the principles of the Declaration of Helsinki. Written informed consent was obtained from the donor's next of kin to use the clinical, pathological, and radiographic data in this study. A total of 73 patients who died from nontraumatic causes in our academic tertiary care hospital and who underwent unenhanced chest antemortem CT, postmortem CT, and pathological autopsy between April 2009 and December 2010 were retrospectively enrolled in this study. Potential donors were excluded for any of the following reasons: (a) age <20 years; (b) treatment with cardiopulmonary resuscitation; (c) diagnosis of congenital heart disease, chronic heart failure, cardiomyopathy, cardiac hypertrophy, or heart amyloidosis; prior cardiovascular surgery; or diagnosis of muscular disorders, such as amyotrophic lateral sclerosis, muscular dystrophy, myositis, or myasthenia. Criterion (c) was confirmed by pathological autopsy. After applying these exclusion criteria, 33 adult human cadavers (22 males, 11 females) were included in this study. The mean age at death was 66 years (range, 21–92 years; median, 72 years). All cadavers were placed in the supine position at room temperature from the time of death until postmortem CT. Antemortem CT was performed at a median of 17 days before death (range, 1–184 days). Postmortem CT was performed at a median of 464 min after death (range, 94–1175 min), followed by pathological autopsy.

### Antemortem CT imaging

Antemortem CT scans were performed for reasons of clinical necessity such as for diagnosis or to ascertain the condition of hospitalized patients. All scans included at least the chest; patients were not excluded from the study if other regions of the body were scanned.

All antemortem CT studies were performed on 64-detector-row helical CT scanners (Aquilion 64, Toshiba Medical Systems Corporation, Ohtawara, Japan; Discovery CT750 HD and LightSpeed VCT, GE Healthcare, Buckinghamshire, UK) in the craniocaudal direction with the patient in the supine position with both arms raised. The scan parameters were as follows: slice thickness, 5 mm; slice interval, 5 mm; rotation time, 0.5 s; and tube voltage, 120 kVp. Automatic tube current modulation was performed by Volume EC (Toshiba) and AutomA (GE). Images were reconstructed at 0.5 mm intervals with a 350 mm field of view and a 512×512 image matrix.

### Postmortem CT imaging

Postmortem CT scans of the whole body were performed to examine the cause of death.

All postmortem CT studies were performed without contrast medium on a 4-detector-row CT scanner (Robusto, Hitachi Medical Corporation, Tokyo, Japan) in helical mode, in the craniocaudal direction. For all scans, the cadaver was laid in the supine position with arms placed on either side of the body. The scan parameters were as follows: slice thickness, 2.5 mm; slice interval, 1.25 mm; rotation time, 0.5 s; tube voltage, 120 kVp; and tube current, 250 mA. Images were reconstructed at 1.25 mm intervals with a 350 mm field of view and a 512×512 image matrix.

### Image interpretation

**Figure 1:**
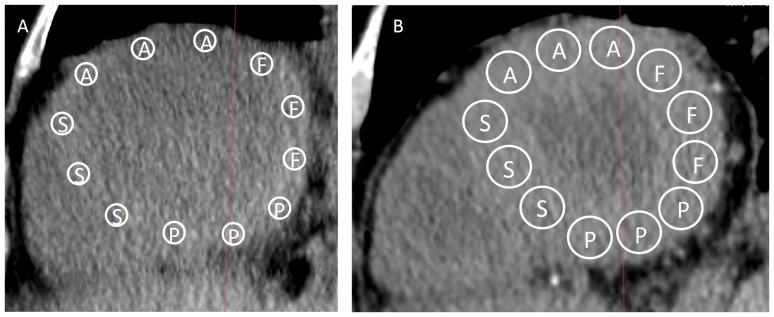
Antemortem and postmortem CT images of the heart in a representative patient. A: Multiplanar reconstruction image obtained by antemortem CT. B: Multiplanar reconstruction image obtained by postmortem CT. Both images were obtained in same plane. A, anterior wall of the left ventricle; F, left ventricular free wall; P, posterior wall of the left ventricle; S, ventricular septum.

**Figure 2:**
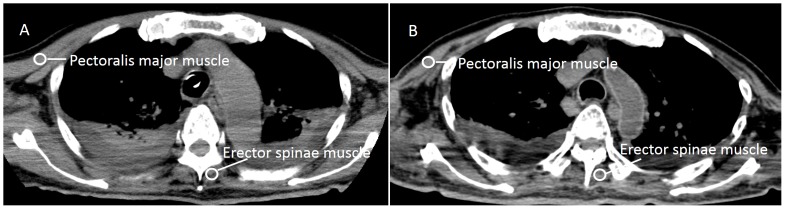
Antemortem and postmortem CT images of the pectoralis major muscle and the erector spinae muscle in a representative patient. A: Antemortem CT. B: Postmortem CT. Both images were obtained at the level of the aortic arch. PMM, pectoralis major muscle; ESM, erector spinae muscle.

All images were interpreted by a radiologist who was not provided with clinical information. The postmortem and most recent antemortem unenhanced chest CT images were compared. CT images were reviewed on a three-dimensional workstation (ZioTerm2009, Ziosoft, Inc., Tokyo, Japan) to obtain multiplanar reconstruction images perpendicular to the line transecting the apex and the center of the mitral valve. A slice at approximately one third of the distance from the apex, corresponding to a pathological section of the heart, was chosen for the analysis. Using the multiplanar reconstruction images, we measured CT attenuation (in Hounsfield units; HU) at three points in each of the following four cardiac muscle sites: anterior wall of the left ventricle, left ventricular free wall, posterior wall of the left ventricle, and the ventricular septum (Fig. 1A,B). The mean values of the three points at each site were calculated. The CT attenuation (in HU) was also determined for the pectoralis major muscle and the erector spinae muscle at the level of the aortic arch (Fig. 2A,B). CT attenuation was measured by setting a circular region of interest (ROI) to the center of each muscle. ROI size was as large as possible without being affected by partial volume phenomena, and the average CT attenuation within the ROI was used for analysis.

### Statistical analyses

We compared the CT attenuation of six sites of striated muscle (i.e., four cardiac muscle sites: anterior wall of the left ventricle, left ventricular free wall, posterior wall of the left ventricle, ventricular septum; and two skeletal muscle sites: pectoralis major muscle and erector spinae muscle) between antemortem CT and postmortem CT using paired *t* tests. Significant differences among the four cardiac muscle sites were determined using Friedman's test. Significant differences between the two skeletal muscle sites were determined using the paired *t* tests. Significant differences between the group of cardiac muscles and that of skeletal muscles were analyzed by unpaired *t* tests. Postmortem change in the CT attenuation of striated muscle was defined as the ratio of CT attenuation of striated muscle on PMCT to that on AMCT. We used the unpaired t-test to analyze postmortem change in the CT attenuation of striated muscle with gender, and linear least squares regression for that with age and elapsed time since death. The level of statistical significance was set at 0.05. Family-wise error was corrected by Bonferroni's correction for each section. All statistical analyses were performed using R version 3.0 (The R Foundation for Statistical Computing, Vienna, Austria; http://www.r-project.org/).

## Results

**Figure 3:**
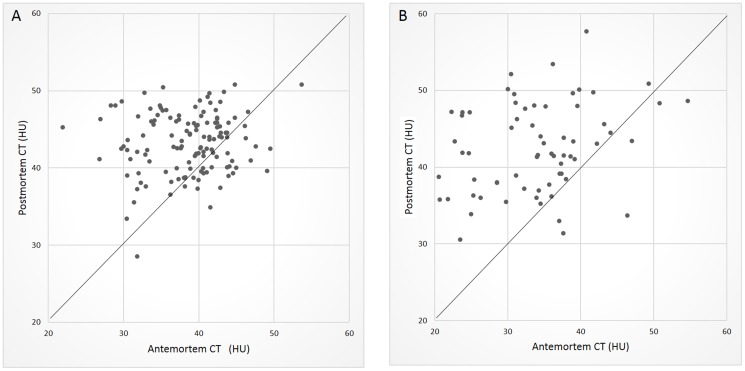
Scatter plot of the CT attenuation values of cardiac and skeletal muscle on antemortem and postmortem CT. A: Cardiac muscle. B: Skeletal muscle.

The CT attenuation values (in HU) of the four cardiac muscle sites and the two skeletal muscle sites on antemortem and postmortem CT are shown in a scatter plot (Fig. 3A,B). These results are also summarized in Table 1. Both cardiac and skeletal muscle showed significantly greater attenuation on postmortem CT than on antemortem CT. The Friedman test showed that there were no significant differences in CT attenuation among the four sites of cardiac muscle either on antemortem or on postmortem CT. There were also no significant differences in CT attenuation between the two sites of skeletal muscle either on antemortem or on postmortem CT. There were significant differences in CT attenuation between the cardiac and skeletal muscle groups on antemortem CT (P<0.001) but not on postmortem CT.

**Table 1:** Comparison of striated muscle attenuation in four cardiac muscle sites, the pectoralis major muscle, and the erector spinae muscle, between antemortem CT and postmortem CT.

Site	Antemortem CT attenuation (HU)	Postmortem CT attenuation (HU)	*P* value*
Cardiac muscle			
Anterior wall of the left ventricle	36.8±6.2	42.5±4.6	<0.001
Left ventricular free wall	38.1±6.2	44.1±3.7	<0.001
Posterior wall of the left ventricle	40.0±3.8	43.8±3.7	<0.001
Ventricular septum	39.6±3.4	42.5±3.2	<0.001
Skeletal muscle			
Pectoralis major muscle	33.7±6.8	43.3±5.9	<0.001
Erector spinae muscle	34.1±8.0	41.7±5.6	<0.001

Values are presented as the mean ± standard deviation.

*Paired *t* tests.

CT, computed tomography; HU, Hounsfield units.

Table 2 summarizes the association of postmortem change in the CT attenuation of striated muscle with gender. Gender showed no statistically significant association with postmortem change in the CT attenuation of striated muscle, at any of the tested sites.

**Table 2:** Association between postmortem change in the CT attenuation of striated muscle and gender.

Site	Male (PM/AM)	Female (PM/AM)	P value
Cardiac muscle			
Anterior wall of the left ventricle	1.1±0.2	1.3±0.3	0.09
Left ventricular free wall	1.1±0.2	1.3±0.2	0.03#
Posterior wall of the left ventricle	1.1±0.1	1.2±0.2	0.04#
Ventricular septum	1.1±0.1	1.1±0.1	0.10
Skeletal muscle			
Pectoralis major muscle	1.3±0.3	1.1±0.3	0.08
Erector spinae muscle	1.4±0.3	1.3±0.3	0.29

Statistical analyses were performed by unpaired t-test.

Values are presented as the mean ± standard deviation.

CT, computed tomography; HU, Hounsfield units; PM/AM, ratio of CT attenuation of striated muscle on postmortem computed tomography to that on antemortem computed tomography.

#Statistically insignificant when family-wise error was corrected by Bonferroni's correction.

Table 3 shows the results of correlation analysis for postmortem change in the CT attenuation of striated muscle with each of age and elapsed time since death. No statistically significant correlation was found at any site.

**Table 3:** Correlation of association between postmortem change in the CT attenuation of striated muscle with age and with elapsed time since death.

Site	Age	Elapsed time since death
Cardiac muscle		
Anterior wall of the left ventricle	0.79	0.12
Left ventricular free wall	0.98	0.34
Posterior wall of the left ventricle	0.30	0.05
Ventricular septum	0.38	0.32
Skeletal muscle		
Pectoralis major muscle	0.09	0.45
Erector spinae muscle	0.51	0.34

Statistical analyses were performed by linear least squares regression.

CT, computed tomography.

## Discussion

Although several studies have evaluated the postmortem changes of the cardiovascular system [12]–[17], few studies have examined the changes in CT attenuation. Shiotani et al. [12] reported that the attenuation of the aortic wall was greater on postmortem CT than on antemortem CT. They also reported that compression of aortic wall components, including collagen fibers, elastic fibers, and smooth muscle, may contribute to the hyperattenuation on postmortem CT. Okuma et al. [15] reported that the heart wall is thicker on postmortem CT than on antemortem CT, which suggests that postmortem contraction of cardiac muscle may cause increase the attenuation of cardiac muscle on postmortem images, as observed in our study.

In the present study, the CT attenuation of the pectoralis major muscle and the erector spinae muscle was significantly greater on postmortem CT than on antemortem CT in the same patients. Lewy et al. [18] reported that postmortem CT did not depict findings specific for rigor mortis, and that rigor did not affect CT attenuation, or the size and shape of skeletal muscles. However, it is well known in the fields of forensic medicine and pathology that rigor mortis causes contraction of striated muscle [19]. Because CT attenuation is affected by the density of muscle [20], the greater attenuation on postmortem CT than on antemortem CT is consistent with the contraction of striated muscle in rigor mortis.

There were no significant intra-group (among the four cardiac muscle sites or between the two skeletal muscle sites) differences in CT attenuation either on antemortem or postmortem CT. Even though they are named differently, the properties of cardiac muscle at different sites in the heart can be quite similar. Regarding the two sites of skeletal muscle, the pectoralis major and erector spinae muscles may also show similar CT attenuation values. Muscles are known to exhibit broad regional differences in muscle fiber density [21], [22] and in CT attenuation [23], [24]. We would have observed a wider variety of CT attenuation values in the group of skeletal muscle if more muscle regions had been added to the analysis.

Antemortem CT attenuation was significantly different between the cardiac and skeletal muscle groups. Because of the marked differences in cardiac and skeletal muscle in terms of their physiological properties as well as their pathological responses, the difference in CT attenuation between these two categories of muscle could reflect these differences in properties. The broad regional differences in muscle fiber density [21], [22] and CT attenuation [23], [24] mentioned earlier mean that the significant differences in antemortem CT attenuation among muscles in different regions detected in this study are to be expected. Although Bulcke et al. [23] and Termote et al. [24] reported similar findings—that the sternocleidomastoideus and iliopsoas are characterized by high attenuation and the gracilis and triceps surae are characterized by low attenuation—there were large differences in the measured CT values for the same muscles. Therefore, we cannot compare the absolute values determined in this study with those of previous studies.

Somewhat unexpectedly, we found no significant differences in postmortem CT attenuation between the cardiac and skeletal muscle groups. One possible explanation is that rigor mortis resulted in marked contraction of the striated muscles, abolishing any possible differences between individual striated muscles on postmortem CT.

If rigor mortis is the principal cause of the hyperattenuation of striated muscle on PMCT, then postmortem change in the CT attenuation of striated muscle should be associated with elapsed time since death, because the presence and degree of rigor mortis generally changes in the period between 1–2 hours and between 24–36 hours after death [25]. In the present study, all PMCT scanning was performed between 1.5 and 20 hours post mortem, which coincides with the timing of rigor mortis. However, our results revealed few associations between postmortem change in the CT attenuation of striated muscle and elapsed time since death. One possibility for this finding is that the total number of patients analyzed in the study was too small to show a positive association.

We also investigated possible confounding factors of postmortem change such as gender and age. The associations between these factors and postmortem change in the CT attenuation of striated muscle were not statistically significant, which indicates that hyperattenuation of striated muscle is a general postmortem finding regardless of gender or age.

Interscanner variability of CT attenuation values (i.e., HU) is an issue that needs to be considered [26], [27], although almost all CT scanners show some intrascanner variability in CT attenuation values [28]. Recent technological innovations, however, may overcome these problems because some studies have shown negligible interscanner variability in terms of CT attenuation values [29]. Although CT attenuation values may not be directly compared between antemortem and postmortem CT because we used different scanners at each time, we fixed the tube voltage at 120 kVp, which should help to reduce the variability. Of course, the significant differences in CT attenuation values between antemortem and postmortem CT cannot be explained without considering postmortem changes if there is any interscanner or intrascanner variability.

Our study has some other limitations. Motion artifacts from the heart may affect antemortem CT but do not affect postmortem CT. It is also possible that underlying diseases, cadaver preservation, atmospheric conditions (e.g. temperature or humidity), or other factors might contribute to the differences observed postmortem [30], [31]. However, some of these factors are unlikely to affect our conclusions because we excluded patients with cardiovascular or muscular diseases from the study, which was confirmed by the pathological autopsy, and all of the cadavers were preserved in accordance with the regulations used at our hospital.

## Conclusions

This is the first longitudinal study to show that striated muscle exhibits significantly greater attenuation on postmortem CT than on antemortem CT in patients who died in hospital from nontraumatic causes.

## References

[1] Thali MJ, Yen K, Schweitzer W, Vock P, Boesch C, et al (2003) Virtopsy, a new imaging horizon in forensic pathology: virtual autopsy by postmortem multislice computed tomography (MSCT) and magnetic resonance imaging (MRI) – a feasibility study. J Forensic Sci 48: 386–403. 12665000

[2] O'Donnell C, Woodford N (2008) Post-mortem radiology - a new sub-speciality? Clin Radiol 63: 1189–1194. 18929036 10.1016/j.crad.2008.05.008

[3] Cha JG, Kim DH, Paik SH, Park JS, Park SJ, et al (2010) Utility of postmortem autopsy via while-body imaging: initial observations comparing MDCT and 3.0 T MRI findings with autopsy findings. Korean J Radiol 11: 395–406. 20592923 10.3348/kjr.2010.11.4.395 PMC2893310

[4] Roberts IS, Benamore RE, Benbow EW, Lee SH, Harris JN, et al (2012) Post-mortem imaging as an alternative to autopsy in the diagnosis of adult deaths: a validation study. Lancet 379: 136–142. 22112684 10.1016/S0140-6736(11)61483-9 PMC3262166

[5] Flach PM, Thali MJ, Germerott T (2014) Times have changed! Forensic radiology - a new challenge for radiology and forensic pathology. Am J Roentgenol 202: W325–W334. 24660730 10.2214/AJR.12.10283

[6] Christe A, Flach P, Ross S, Spendlove D, Bolliger S, et al (2010) Clinical radiology and postmortem imaging (Virtopsy) are not the same: Specific and unspecific postmortem signs. Leg Med (Tokyo) 12: 215–222. 20630787 10.1016/j.legalmed.2010.05.005

[7] Shiotani S, Kohno M, Ohashi N, Yamazaki K, Nakayama H, et al (2004) Non-traumatic postmortem computed tomographic (postmortem CT) findings of the lung. Forensic Sci Int 139: 39–48. 14687772 10.1016/j.forsciint.2003.09.016

[8] Ishida M, Gonoi W, Hagiwara K, Takazawa Y, Akahane M, et al (2011) Intravascular gas distribution in the upper abdomen of non-traumatic in-hospital death cases on postmortem computed tomography. Leg Med (Tokyo) 13: 174–179. 21561795 10.1016/j.legalmed.2011.03.002

[9] Ishida M, Gonoi W, Hagiwara K, Takazawa Y, Akahane M, et al (2011) Hypostasis in the heart and great vessels of non-traumatic in-hospital death cases on postmortem computed tomography: relationship to antemortem blood tests. Leg Med (Tokyo) 13: 280–285. 22000062 10.1016/j.legalmed.2011.09.004

[10] Ishida M, Gonoi W, Hagiwara K, Takazawa Y, Akahane M, et al (2011) Postmortem changes of the thyroid on computed tomography. Leg Med (Tokyo) 13: 318–322. 21940188 10.1016/j.legalmed.2011.08.003

[11] Ishida M, Gonoi W, Hagiwara K, Okuma H, Shintani Y, et al (2014) Fluid in the airway of nontraumatic death on postmortem computed tomography: relationship with pleural effusion and postmortem elapsed time. Am J Forensic Med Pathol 35: 113–117. 24781399 10.1097/PAF.0000000000000083

[12] Shiotani S, Kohno M, Ohashi N, Yamazaki H, Nakayama H, et al (2002) Hyperattenuating aortic wall on postmortem computed tomography (postmortem CT). Radiat Med 20: 201–206. 12296437

[13] Shiotani S, Kohno M, Ohashi N, Yamazaki K, Nakayama H, et al (2003) Dilation of the heart on postmortem computed tomography (postmortem CT): comparison with live CT. Radiat Med 21: 29–35. 12801141

[14] Hyodoh H, Sato T, Onodera M, Washio H, Hasegawa T (2012) Vascular measurement changes observed using postmortem computed tomography. Jpn J Radiol 30: 840–845. 23054882 10.1007/s11604-012-0134-z

[15] Okuma H, Gonoi W, Ishida M, Shintani Y, Takazawa Y, et al (2013) Heart wall is thicker on postmortem computed tomography than on ante mortem computed tomography: the first longitudinal study. PLoS One 8: e76026. 24086680 10.1371/journal.pone.0076026 PMC3785517

[16] Okuma H, Gonoi W, Ishida M, Shintani Y, Takazawa Y, et al.. (2013) Greater thickness of the aortic wall on postmortem computed tomography compared with antemortem computed tomography: the first longitudinal study. Int J Legal Med In press; doi: 10.1007/s00414-013-0955-z. 10.1007/s00414-013-0955-z 24352561

[17] Takahashi N, Higuchi T, Hirose Y, Yamanouchi H, Takatsuka H (2013) Changes in aortic shape and diameters after death: comparison of early postmortem computed tomography with antemortem computed tomography. Forensic Sci Int 225: 27–31. 22656269 10.1016/j.forsciint.2012.04.037

[18] Lewy AD, Harcke HT, Mallak CT (2010) Postmortem imaging: MDCT features of postmortem change and decomposition. Am J Forensic Med Pathol 31: 12–17. 20010292 10.1097/PAF.0b013e3181c65e1a

[19] Smith RD (1950) Studies on rigor mortis, Part 2, Qualitative observations on the post mortem shortening of muscles. Anat Rec 108: 207–216. 14799868 10.1002/ar.1091080203

[20] Mull RT (1984) Mass estimates by computed tomography: physical density from CT numbers. AJR Am J Roentgenol 143: 1101–1104. 6333158 10.2214/ajr.143.5.1101

[21] Hadar H, Gadoth N, Heifetz M (1983) Fatty replacement of lower paraspinal muscles: normal and neuromuscular disorders. AJR Am J Roentgenol 141: 895–898. 6605058 10.2214/ajr.141.5.895

[22] Hawley RJ Jr, Schellinger D, O'Doherty DS (1984) Computed tomographic patterns of muscles in neuromuscular diseases. Arch Neurol 41: 383–387. 6703939 10.1001/archneur.1984.04050160045014

[23] Bulcke JA, Termote JL, Palmers Y, Crolla D (1979) Computed tomography of the human skeletal muscular system. Neuroradiology 17: 127–136. 450236 10.1007/BF00339869

[24] Termote JL, Baert A, Crolla D, Palmers Y, Bulcke JA (1980) Computed tomography of the normal and pathologic muscular system. Radiology 137: 439–444. 7433677 10.1148/radiology.137.2.7433677

[25] Tsokos M (2005) Postmortem changes. In: Payne-James J, Byard RW, Corey TS and Henderson C, editors.Encyclopedia of forensic and legal medicine.Oxford: Elsevier Academic Press. pp.456–476.

[26] Levi C, Gray JE, McCullough EC, Hattery RR (1982) The unreliability of CT numbers as absolute values. AJR Am J Roentgenol 139: 443–447. 6981306 10.2214/ajr.139.3.443

[27] Groell R, Rienmueller R, Schaffler GJ, Portugaller HR, Graif E, et al (2000) CT number variations due to different image acquisition and reconstruction parameters: a thorax phantom study. Comput Med Imaging Graph 24: 53–58. 10767584 10.1016/s0895-6111(99)00043-9

[28] Birnbaum BA, Hindman N, Lee J, Babb JS (2007) Multi-detector row CT attenuation measurements: assessment of intra- and interscanner variability with an anthropomorphic body CT phantom. Radiology 242: 109–119. 17185663 10.1148/radiol.2421052066

[29] Nishihara S, Koike M, Ueda K, Sanada T, Ebitani K, et al (2002) Intra- and inter-equipment variations in the mean CT numbers of a vertebral body for X-ray CT equipment. Med Imag Inform Sci 20: 40–43 (in Japanese)..

[30] Jackowski C, Sonnenschein M, Thali MJ, Aghayev E, Yen K, et al (2007) Intrahepatic gas at postmortem computed tomography: forensic experience as a potential guide for in vivo trauma imaging. J Trauma 62: 979–988. 17426557 10.1097/01.ta.0000198733.22654.de

[31] Singh MK, O'Donnell C, Woodford NW (2009) Progressive gas formation in a deceased person during mortuary storage demonstrated on computed tomography. Forensic Sci Med Pathol 5: 236–242. 19669956 10.1007/s12024-009-9103-y

